# Teaching Simple Strategies to Foster Emotional Well-Being

**DOI:** 10.3389/fpsyg.2021.772260

**Published:** 2021-11-10

**Authors:** Emily A. Iovino, Jessica B. Koslouski, Sandra M. Chafouleas

**Affiliations:** ^1^Collaboratory on School and Child Health, Institute for Collaboration on Health, Intervention, and Policy, University of Connecticut, Storrs, CT, United States; ^2^Department of Educational Psychology, Neag School of Education, University of Connecticut, Storrs, CT, United States

**Keywords:** emotional well-being, kernels, school-based strategies, coping strategies, positive emotion, cognitive behavioral therapy

## Abstract

The COVID-19 pandemic has presented considerable disruptions to routines that have challenged emotional well-being for children and their caregivers. One direction for supporting emotional well-being includes strategies that help children feel their best in the moment, which can bolster their capacity to respond appropriately to thoughts and behaviors. Strengthening emotional well-being equitably, however, must include opportunities in settings that are easily accessible to all, such as schools. In this paper, we focus on simple, evidence-informed strategies that can be used in schools to promote positive feelings in the moment and build coping behaviors that facilitate tolerance of uncertainty. We focus on those strategies that educators can easily and routinely use across ages, stages, and activities. Selected strategies are primarily tied to cognitive behavioral theory, with our review broadly organized across categories of self-awareness, self-soothing, and social relationships. We review evidence for each, providing examples that illustrate ease of use in school settings.

## Introduction

Though definitions of emotional well-being (EWB) vary (Center for Disease Control and Prevention, 2018; Feller et al., 2018; National Institute of Health, 2021), it is generally agreed that EWB is comprised of multiple dimensions that reflect how an individual feels in the moment, generally, and about life. Our life events and experiences (e.g., language, art/music, noises, and faces) can be placed along an emotion continuum from positive to negative affective quality. This continuum in which a stimulus is felt as pleasing or displeasing is referred to as emotional valence. When we feel our best self, our emotional valence is generally positive. Emotional valence is critical for representing and categorizing human experiences and serves to influence behavior and cognition (Kauschke et al., 2019). As such, becoming aware of feeling in the moment – such as paying attention to physical sensations of emotional states – is key to changing thoughts and behaviors. In fact, interventions designed to increase positive emotion have been shown to increase behavioral and cognitive repertoires (Isen, 1987; Fredrickson and Branigan, 2005; Fredrickson et al., 2008; Schutte, 2014). Researchers have suggested that optimal emotional well-being occurs when positive affect is experienced by individuals at rates three times more than negative (Diehl et al., 2011). In addition, for those exposed to chronic stress, engaging in strategies that evoke positive emotion can disrupt the accumulation of, and sensitization to, chronic stress. Thus, identifying how to invoke positive emotion in the moment and sustain it over time is critical to well-being (Sheldon and Lyubomirsky, 2006). In this paper, we focus on simple strategies that can be used in schools to promote positive feelings in the moment and serve to build coping behaviors to facilitate tolerance of uncertainty.

### Strategies to Promote EWB

Cognitive behavioral therapy (CBT) is a commonly used intervention framework for mental health intervention to promote EWB (Early and Grady, 2017). CBT draws from both cognitive and behavioral theory, each of which brings a different focus. Cognitive theory centers around how thought impacts emotions and behavior, and behavioral theory focuses on how factors in the environment cue and reinforce behavior (Early and Grady, 2017). The interplay of the two theories situates a person and their internal world within the external environment, acknowledging how both internal and external factors influence an individual’s behavior. CBT practice models go beyond the integration of cognitive and behavioral theories to include focus on the multidirectional influence of affect (emotion), behavior, and cognition (thought; Early and Grady, 2017). Given this multidirectional interplay, intervention strategies are therefore focused on changing emotions, behaviors, or thoughts as changing one aspect will influence the others.

CBT is used as the foundation for many interventions that seek to promote EWB in U.S. schools. For example, a recent systematic review found that a majority of resilience-building interventions in schools were based on CBT (Dray et al., 2017). The popularity of CBT may be due to the established effectiveness as an intervention (Benjamin et al., 2011). A recent review of meta-analyses and systematic reviews, for example, reported small to medium effect sizes for the effectiveness of short-term school-based CBT programs for anxiety prevention and resilience building, such as the *FRIENDS* program (Šouláková et al., 2019). We acknowledge that CBT is just one of many therapeutic approaches but focus on it due to its predominance in existing school interventions.

Although school-based CBT programs are effective, there are challenges when attempting to disseminate these programs “widely and effectively” in schools in ways that can benefit all children (Embry and Biglan, 2008, p. 75). Although universal mental health interventions in schools have begun to integrate CBT-based approaches (Dray et al., 2017), these interventions are typically targeted to children requiring support for a specific diagnosis or concern as opposed to proactive implementation as a promotion strategy for the general population (Creed et al., 2016). In addition, implementing a school-based CBT program at a large scale and maintaining fidelity can be challenging given resources (e.g., dedicated staff, time, and financial investment) necessary to put in place in a way that can universally impact well-being (Embry and Biglan, 2008). Having classroom teachers deliver CBT interventions could maximize access to universal strategies (Forman and Barakat, 2011), yet available intervention protocols in the United States often require advanced training to build the knowledge and skills to enable successful implementation.

Given estimates suggesting a doubling of rates of anxiety and depression among children and adolescents during the global pandemic (Racine et al., 2021), these challenges to implementing universal well-being promotion in schools are particularly concerning in pandemic. Social isolation and disruption to routines have been noted as contributors to the increased prevalence of mental health challenges, with tolerance for uncertainty identified as a predictor of anxiety and depression among adults during the COVID-19 pandemic (Rettie and Daniels, 2021). Tolerance for uncertainty refers to ability to engage in positive coping behaviors during times of uncertainty. Given the importance of tolerance for uncertainty, schools need capacity to implement universal strategies that can facilitate positive coping behaviors.

Promoting tolerance for uncertainty does not necessitate complex and intensive intervention packages, with adaptive coping involving use of tools such as humor and social connection. Along these lines, child and adolescent intervention researchers have been working to identify “active ingredients,” that is, those core components of intervention that lead to behavioral change. Active ingredients in intervention for youth with anxiety and depression, for example, include strategies such as behavioral activation, physical activity, mental imagery, and social relationships (Wolpert et al., 2021). In a similar vein, Embry and Biglan (2008) proposed the term “kernels” to refer to these fundamental units of behavior influence (77), with recommendations to extend use beyond treatment to universal promotion and prevention of well-being. Their review identified common elements of evidence-based programs, referring to each strategy as an evidence-based kernel. Examples of evidence-based kernels include nasal breathing, self-monitoring, and praise (Embry and Biglan, 2008; Chafouleas et al., 2020).

In this paper, we focus on kernels, or active ingredients, of CBT-based interventions that can be proactively and easily used in schools to promote EWB. Wolpert et al. (2021) identified active ingredients in intervention for youth with anxiety and depression within six broad groups (see Table 1). We organize our paper using three broader categories of active ingredients: self-awareness (e.g., psychoeducation and cognitive restructuring), self-soothing (e.g., relaxation strategies), and social relationships. We conceptualize these categories as capturing each of Wolpert et al.’s categories that can be addressed in schools; this alignment between can be found in Table 1. Our selected categories reference simple strategies that educators can easily and routinely use across ages, stages, and settings to enable children to feel their best self in the moment and build positive coping behaviors.

**Table 1:** A crosswalk of our categories and Wolpert et al.’s (2021) categories of active ingredients.

Wolpert et al.’s (2021) Categories of Active Ingredients	Our Categories of Simple Strategies		
	Self-Awareness	Self-Soothing	Social Relationships
Behaviors and Activities		X	X
Beliefs and Knowledge	X		
Brain and Body Functions		X	
Cognitive and Attentional Skills	X	X	
Human Connections			X
Socioeconomic Factors			

An “X” indicates overlap between our categories and Wolpert et al.’s (2021) active ingredients. Examples of “socioeconomic factors” as defined by Wolpert et al. (2021) include economic transfers and access to green space. Given that we are focused on simple school-based strategies, this is not explicitly addressed in our categories.

Next, we review evidence behind these three categories and highlight simple strategies within each that promote positive feeling in the moment. We offer examples to adapt the simple strategies to be universally accessed by children across ages and easily embedded within the learning environment. See Table 2 for a summary.

**Table 2:** Simple strategies to promote child self-awareness, self-soothing, and social relationships.

Category	Simple Strategy	Example Adaptation
Self-Awareness	Psychoeducation	Mini-lessons that can be integrated into academics (e.g., reading, writing) on how to recognize emotions in oneself and in others
	Cognitive Restructuring (e.g., affirmations, acknowledgments)	Recurring brief activities that teach shifting unpleasant thoughts to more pleasant thoughts
Self-Soothing	Breathing techniques	Belly breathing, in which a child breathes in deeply to inflate their stomach, holds the breath, and then exhales to deflate their stomach
	Movement	Allowing opportunities for physical activity throughout the day
	Visualization	Before a math test, walking the class through an exercise in which they visualize their success on the assessment
	Stretching	5-min yoga practice after returning from recess
	Progressive Muscle Relaxation	Using a script, teacher leads the class through tightening and releasing muscle groups
Social Relationships	Gratitude	During art class, children create a drawing to give to a teacher or classmate for whom they are grateful
	Acts of Kindness	Have children create “tootle” notes, in which they write compliments to a peer
	Seeking Social Support	5-min lesson during morning meeting about when and how to share their feelings with a trusted individual

## Self-Awareness

Definitions of self-awareness may emphasize paying attention to oneself to facilitate self-knowledge and enable awareness and evaluation of thoughts and feelings or may focus on awareness of one’s own internal states and how these states are influenced by others and one’s environment (Feize and Faver, 2019), and associated strategies promote awareness of one’s thoughts and feelings, understanding of how thoughts and feelings influence behavior, and awareness of how one’s thoughts, feelings, and behavior impact themselves and others.

Self-awareness has shown to be important for enhancing EWB. For one, a lack of awareness of one’s own emotions has been associated with increased likelihood of internalizing symptoms and, ultimately, development of internalizing disorders (Sendzik et al., 2017; Van Beveren et al., 2019). Low emotional awareness has also been associated with more externalizing problems, particularly for children with attention-deficit/hyperactivity disorder (Factor et al., 2016). For youth, better emotional self-awareness has shown to predict adaptive emotional regulation (Van Beveren et al., 2019).

As shown in Table 2, one simple strategy to promote self-awareness is psychoeducation. Psychoeducation is well-established as an effective intervention and has been associated with positive outcomes for children and adolescents (Lukens and McFarlane, 2004; Tanaka et al., 2020; Noble et al., 2021). Although there are many conceptualizations of and ways to deliver psychoeducation, strategies broadly involve educating an individual about their own emotional states, including internal and external influences of these states and coping strategies that promote adaptive functioning. For example, psychoeducation as a simple strategy might include mini-lessons that can be integrated into academic subjects, such as reading and writing, on how to recognize emotions in oneself and others (e.g., what it looks and feels like to be sad, happy, angry). Another strategy involves cognitive restructuring, which is a key component of CBT-based intervention packages. Cognitive restructuring strategies teach individuals to identify, evaluate, and shift unhelpful thoughts (Clark, 2013). Two examples of cognitive restructuring are affirmations (i.e., positive statements about one’s self-worth; (Nelson et al., 2014) and acknowledging (i.e., reminding oneself of previous success). As a simple strategy, cognitive restructuring might look like recurring brief activities (e.g., 5-min lessons before introducing new academic concepts) that teach and reinforce how to shift overwhelmed or frustrated thoughts to be more positive. For example, a teacher could ask children to remember a time when they were successful with a challenging task. Then, teachers can discuss how when feeling overwhelmed with a new challenging task, students can think about their previous success to shift their thoughts to be more positive. Through psychoeducation and cognitive restructuring strategies, an individual can better understand their thoughts and feelings, appropriate responses to positively or negatively valanced situations, and strategies for shifting their thoughts.

## Self-Soothing

The acquisition of self-soothing skills begins in early childhood (e.g., thumb sucking and rocking) and can be taught and learned across the life course (Murray et al., 2015). Self-soothing refers to an individual’s efforts or ability to calm themselves when emotionally distressed and during the resultant autonomic nervous system arousal (Wright, 2009). Emotional distress can result from feelings of fear, embarrassment, or anger. When individuals are emotionally distressed, the nervous system perceives threat and works to protect the individual (i.e., activating the sympathetic nervous system or “fight or flight”; Kozlowska et al., 2015). Common nervous system responses are increased heart and breathing rates, tightened muscles in preparation for self-defense, and narrowed attentional focus on the source of distress (Perry et al., 1995; Gable and Harmon-Jones, 2010; Kozlowska et al., 2015). In addition, the neocortex, the area of the brain responsible for higher-order thinking (e.g., planning and inhibition), becomes less active (Perry et al., 1995).

When individuals are distressed, they have minimal cognitive energy to devote to new tasks (Perry et al., 1995). Therefore, self-soothing activities focus on calming the nervous system (Wright, 2009). These activities can soothe by helping to slow rapid heart rate and breathing and relax muscles. They also shift attentional focus from the source of distress, which can be particularly important for individuals to regain a sense of calm. When distressed, shifting attentional focus can be facilitated through strategies that engage the senses (Linehan, 1993) or rhythmic and repetitive movement (Perry, 2009). Rhythm and repetition calm the nervous system by restoring bodily rhythms that promote a sense of safety. Self-soothing kernels facilitate the reactivation of the parasympathetic nervous system (i.e., “rest and digest”), allowing individuals to calm from distressed states and reengage with the broader environment. Positive self-soothing skills have been associated with improved emotional regulation, social interactions with peers, and on-task behavior (Wyman et al., 2010).

Self-soothing skills are important in school environments, helping children to respond to distressing situations (e.g., embarrassment or rejection and feelings of failure or incompetence) and return to learning and optimal levels of engagement with the environment (Cicchetti et al., 1991). Importantly, self-soothing skills can be taught and practiced when individuals are calm and regulated. This allows for processing and learning of the strategies and the development of habits that can be subsequently used during times of emotional distress. In addition, self-soothing skills that are embedded into daily routines (e.g., regularly beginning classroom lessons with children taking five deep breaths) are more likely to be practiced (National Academies of Sciences, Engineering, and Medicine, 2019). Strategies, including breathing techniques, movement, visualization, stretching, and progressive muscle relaxation, facilitate self-soothing. In Table 2, example universal adaptations for each of these simple strategies are provided. For example, breathing techniques as a simple strategy might look like teaching belly breathing to all students, where they breathe in to inflate the stomach, hold for 5seconds, and release the breath to deflate the stomach.

## Social Relationships

Social relationships are trusted connections in which support is given or received. Early in the life course, infant and young children’s closest social relationships are typically with their caregivers and close family members. As children begin to engage with childcare or schooling, these relationships often grow to include teachers, peers, coaches, and other important adults in their lives. Across the life course, positive social relationships are key to healthy development and EWB (Osher et al., 2020).

Positive social relationships have many positive effects on children and classrooms. When met with validating and patient responses, positive social interactions help children process and respond to their feelings (Thompson and Meyer, 2007). In addition, they may foster a sense of emotional closeness and reinforce feelings of trust between the child and teacher, parent, guardian, or friend (Buhrmester, 1990; Shulman et al., 1997; Thompson and Meyer, 2007; Brown, 2013). For example, positive child-teacher relationships promote self-regulation, which can improve classroom behavior and classroom climate (Osher et al., 2020). Teachers’ efforts to build strong, quality relationships with children are a priority. This requires intentional effort and can be challenging with children displaying disruptive or withdrawn behaviors or in middle and high school contexts where teachers typically work with many children for shorter periods of time (i.e., due to rotating classes and semester scheduling). However, it is particularly important in these contexts: positive relationships with others can buffer and repair areas of the brain impacted by trauma (National Academies of Sciences, Engineering, and Medicine, 2019), and relationships with competent and caring adults are critical for brain and psychosocial development during adolescence (Immordino-Yang et al., 2018; Osher et al., 2020).

Social relationships can be cultivated through expressions of gratitude, offering kindness in words or actions, and seeking social support. These strategies within the social relationships category are outlined in Table 2. Literature reviews and metanalyses find that gratitude is strongly associated with social relationships, pro-social emotions, and well-being across the life course (Wood et al., 2010; Ma et al., 2017). Gratitude interventions can be particularly beneficial for children and adolescents with lower levels of positive affect (Froh et al., 2009) and have been associated with improved school satisfaction (Froh et al., 2008). Gratitude as a simple strategy might look like having children create a special drawing to give to an adult or peer for whom they are grateful. Relatedly, acts of kindness – where children offer kind words or actions to others – have been found to be highly beneficial for the *giver* in addition to the receiver. For example, performing acts of kindness is associated with increased happiness in young children (Aknin et al., 2012) as well as increased well-being and peer acceptance in elementary school children (Layous et al., 2012). Importantly, performing acts of kindness has stronger effects for the giver when they provide opportunities for social connection (Aknin et al., 2012). As an example of acts of kindness as a simple strategy, children may be instructed to create “tootle” notes for peers during morning meeting. Finally, seeking emotional support, in which children share feelings (e.g., sadness, nervousness) with a trusted teacher, parent, guardian, or friend, can foster social relationships and improve EWB (Umberson and Montez, 2010). For example, as a simple strategy, a child might be taught by a caregiver when and how to express their emotions to a trusted individual.

## Discussion

In this article, we reviewed evidence for the importance of self-awareness, self-soothing, and social relationships for universal promotion of EWB in child populations. We summarized simple strategies within each category, sharing how each can be used by educators to promote EWB by facilitating adaptive coping strategies and strengthening tolerance for uncertainty.

Educators need simple strategies in their everyday toolbox for several reasons. First, they are taxed for time and resources, needing to teach a great deal of content and skills to children over the course of each school year. Barriers to existing SEB curricula include cost, time required for teaching content, and staff training requirements (Embry and Biglan, 2008; Creed et al., 2016). Relatedly, though high-quality professional development is needed to deliver current SEB curricula (National Academies of Sciences, Engineering, and Medicine, 2019), implementation remains scarce, and the quality and duration of this training vary widely (Jennings and Frank, 2015). Second, there is a need to promote EWB and increase tolerance for uncertainty on a universal level, especially in present times due to the COVID-19 pandemic. Therefore, we have amplified examples of simple strategies that educators can implement universally with minimal training, in short periods of time (e.g., 1–2min during a transition), and at little or no cost. In this way, we have matched strategies to the landscape of teacher knowledge and competing demands in schools. These strategies allow educators to prioritize universal SEB needs quickly and efficiently without sacrificing other domains.

Future research is needed to curate and disseminate evidence-based simple strategies that can be easily adapted and incorporated for use in schools. Important questions remain about how such a collection might be presented to promote effective and efficient implementation, how to tailor the strategies for a range of developmental ages and profiles, and whether certain strategies (e.g., breathing techniques) have differential benefits for certain populations or conditions. Finally, research is needed to understand the malleability of children’s affective dispositions. Though some suggest that individuals have a “happiness set point,” or average level of happiness, others have found that adult coping skills are malleable (Rettie and Daniels, 2021), which has interesting implications for research with child populations.

## Conclusion

In conclusion, child EWB has important implications for success in school and life. We have categorized evidence and curated simple strategies in three broad categories: self-awareness, self-soothing skills, and social relationships. Simple strategies can offer a way for educators to embed these important skills into the school day to promote EWB and child success in their classrooms. Strategies that promote positive coping and increase tolerance for uncertainty are especially critical amid extreme uncertainty resulting from the current COVID-19 pandemic.

## Author Contributions

All authors listed have made a substantial, direct and intellectual contribution to the work and approved it for publication.

## Funding

The authorship and publication of this article were made possible in part by funding from the Neag Foundation, which serves as a philanthropic force for positive change in education, health, and human services initiatives.

## Conflict of Interest

The authors declare that the research was conducted in the absence of any commercial or financial relationships that could be construed as a potential conflict of interest.

## Publisher’s Note

All claims expressed in this article are solely those of the authors and do not necessarily represent those of their affiliated organizations, or those of the publisher, the editors and the reviewers. Any product that may be evaluated in this article, or claim that may be made by its manufacturer, is not guaranteed or endorsed by the publisher.
